# SARS-CoV-2 infection among physicians over time in Ontario, Canada: a population-based retrospective cohort study

**DOI:** 10.3325/cmj.2024.65.30

**Published:** 2024-02

**Authors:** Steven Habbous, Natasha Saunders, Kelvin KW Chan, Susy Hota, Jonathan Wang, David Messenger, Erik Hellsten

**Affiliations:** 1 Ontario Health, Strategy, Planning, and Analytics, Ontario, Canada; 2 The Hospital for Sick Children, Toronto, Ontario, Canada; 3 Sunnybrook Health Sciences Centre, Odette Cancer Centre, Toronto, Ontario, Canada; 4 Division of Infectious Diseases, Department of Medicine, University Health Network, Toronto, Ontario, Canada; 5 Department of Critical Care, Queen's University, Kingston, Ontario, Canada

## Abstract

**Aim:**

To assess this risk of SARS-CoV-2 infection among Ontario physicians by specialty and in comparison with non-physician controls during the COVID-19 pandemic.

**Methods:**

In this retrospective cohort study, the primary outcome was incident SARS-CoV-2 infection confirmed by polymerase chain reaction (PCR). Secondary outcomes were hospitalization, use of critical care, and mortality.

**Results:**

From March 1, 2020 to December 31, 2022, 6172/30 617 (20%) active Ontario physicians tested positive for SARS-CoV-2. Infection was less likely if physicians were older (OR 0.78 [0.76-0.81] per 10 years), rural residents (OR 0.70 [0.59-0.83]), and lived in more marginalized neighborhoods (OR 0.74 [0.62-0.89]), but more likely if they were female (OR 1.14 [1.07-1.22]), worked in long-term care settings (OR 1.16 [1.02-1.32]), had higher patient volumes (OR 2.05 [1.82-2.30] for highest vs lowest), and were pediatricians (OR 1.25 [1.09-1.44]). Compared with community-matched controls (n = 29 763), physicians had a higher risk of infection during the first two waves of the pandemic (OR 1.38 [1.20-1.59]) but by wave 3 the risk was no longer significantly different (OR 0.93 [0.83-1.05]). Physicians were less likely to be hospitalized within 14 days of their first positive PCR test than non-physicians (*P* < 0.0001), but there was no difference in the use of critical care (*P* = 0.48) or mortality (*P* = 0.15).

**Conclusion:**

Physicians had higher rates of infection than community-matched controls during the first two waves of the pandemic in Ontario, but not from wave 3 onward. Physicians practicing in long-term care facilities and pediatricians were more likely to test positive for SARS-CoV-2 than other physicians.

During the severe acute respiratory syndrome (SARS) outbreak in 2003 in Toronto, Ontario, patients admitted to hospital with SARS became a source of infection and psychological stress for hospital staff (1,2). This experience culminated in hard lessons learned about the role of containment and the use of personal protective equipment during an outbreak (3).

Despite these lessons, the SARS-CoV-2 pandemic that emerged in 2019 presented a different set of challenges owing to its long duration and changing transmissibility and virulence. These factors, combined with global supply chain issues (eg, personal protective equipment) and uncertainty around the risk of infection, the COVID-19 pandemic has taken a substantial and sustained toll on the mental health of health care staff (4-8).

Physicians work in high-risk settings (some more than others) and have limited ability to work remotely. They are therefore at risk of contracting SARS-CoV-2 by virtue of their employment. Despite this, information about SARS-CoV-2 positivity among physicians over time and by specialty is lacking, with the majority of studies being limited to the first year of the pandemic (9-12). Following the first year, changes in masking practices, public space restrictions, return to in-person learning in schools, fatigue among health care workers, and the introduction of more infectious variant strains may have contributed to changing patterns of infection (13-15). Thus, a more contemporary analysis of trends is needed. In the present study, we estimated the rate of incident SARS-CoV-2 infection, hospitalization, critical care use, and mortality among physicians by specialty and across multiple waves of SARS-CoV-2 variants in Ontario, Canada. Results from this study can be used to inform strategies that keep physicians in the workplace safe from infection during future outbreaks.

## Methods

### Physician cohort creation

This was a retrospective cohort study of physicians and non-physician controls. Active physicians were identified from the Ontario Health Insurance Program (OHIP) database by capturing all unique physician billing numbers between March 1, 2020 and December 31, 2022. OHIP records were extracted on May 30, 2023. The physician billing number was used to link to the Corporate Provider Database (CPDB) to obtain the physicians’ first name, middle name, surname, and city and postal code of employment, restricted only to workplaces that were not expired before March 1, 2020.

**Figure 1:**
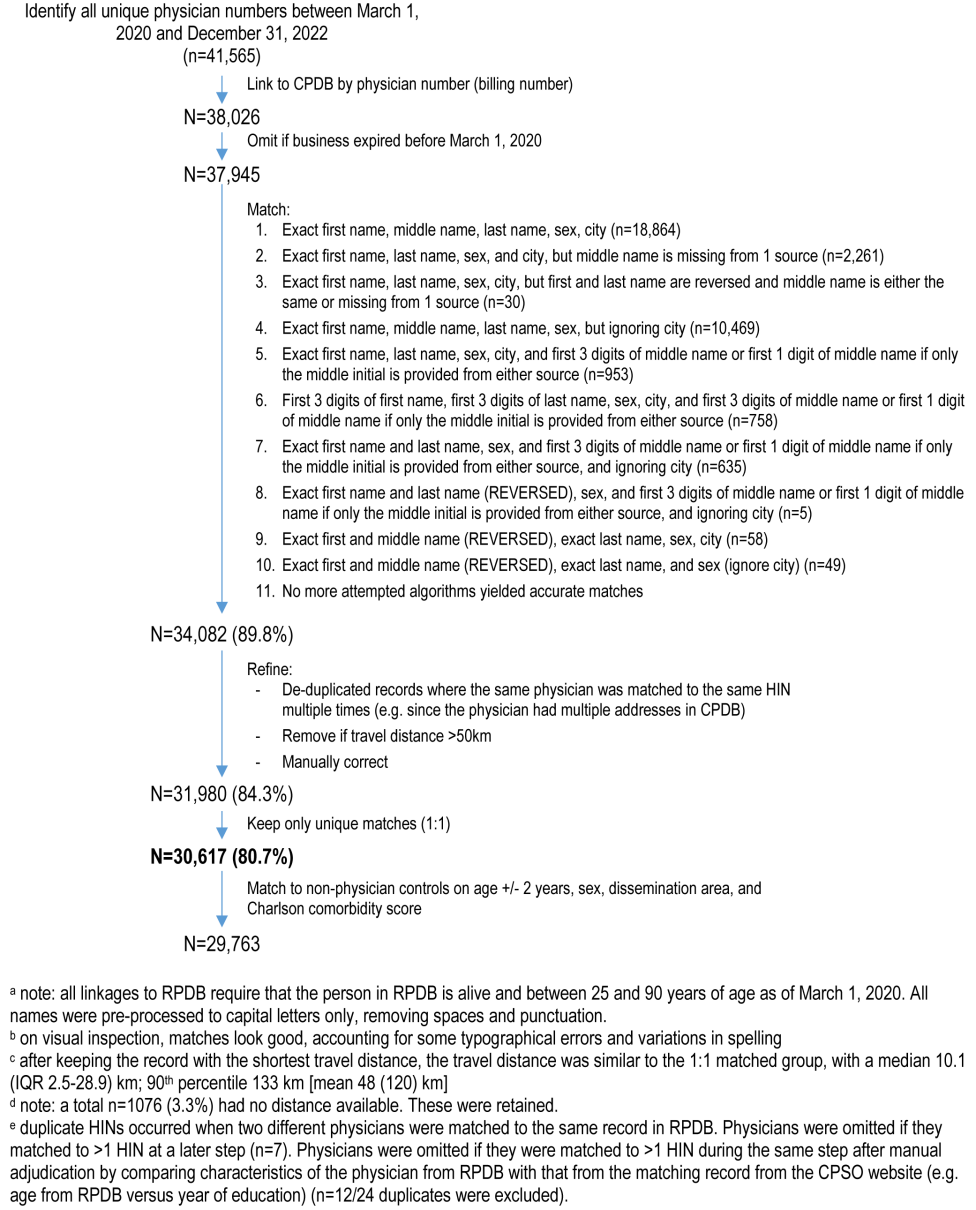
Physician cohort creation. Data sources and strategy to identify physicians from health administrative databases. RPDB – Registered Persons Database; CPDB – Corporate Provider Database; HIN – health insurance number (unique person identifier); CPSO - College of Physicians and Surgeons of Ontario. When the first and last name were reversed, for example, this means the first name from RPDB was matched to the last name from CPDB and the last name from RPDB was matched to the first name from CPDB.

To obtain physicians’ health card numbers, we linked to the Registered Persons Database (RPDB) using various combinations of name and geography (Figure 1; Supplemental Table 1(Supplementary table 1)). The straight-line distance between the postal code of residence (RPDB) and employment (CPDB) was computed by using the latitude and longitude of the respective postal codes. To improve the quality of linkage, we restricted the pool of Ontarians to those between the ages of 25 and 80 years (as of March 1, 2020) and within a reasonable distance (50 km travel distance) of their reported closest place of employment. The 50-km cut-off point was chosen *ad hoc* after looking at the distribution of travel distances for the best matches (1:1 matches on exact first name, middle name, surname, sex, and city) (n = 18 864; Figure 1). Only unique matches were kept, resulting in 30 617 of 37 945 physicians being linked (80.7% match rate) and available for further linkage and analysis.

### Control cohort creation

For comparison with physicians, we identified two control groups. The community-matched non-physician control group were Ontarians matched 1:1 to physicians on age ±2 years, sex, Charlson comorbidity score, and small-area geography (dissemination area). The Charlson comorbidity score was estimated by using a 3-year lookback window from March 1, 2020, categorized as 0 (no comorbidity), 1 comorbidity, 2 comorbidities, and 3+ comorbidities. Dissemination areas include, on average, approximately 600 residents and are the smallest analytic units of geography available. The general non-physician control group included a random sample of all non-physician Ontario residents between 25 and 80 years of age as of March 1, 2020, frequency-matched to the physicians’ distribution of age-sex strata. Age strata were 25-35, 36-45, 46-65, and 66-80 years. Characteristics of the matched physicians, community controls, and general population controls were similar on matched factors (Supplemental Table 2(Supplementary table 2)).

### Covariates

Physician specialty was captured from the CPDB. We classified physicians as working at a long-term care (LTC) institution if they billed at least 20 instances between March 1, 2020 and March 1, 2021 with a billing code starting with “W,” corresponding to services for patients in chronic care hospitals, convalescent hospitals, nursing homes, homes for the aged, and designated chronic or convalescent care beds in hospitals.

### Outcomes

Using health card numbers, we linked to the Ontario Laboratories Information System COVID-19 database to ascertain SARS-CoV-2 infection confirmed by the polymerase chain reaction (PCR) test, which captures >90% of all cases in the province (16). Only positive SARS-CoV-2 PCR tests were included in the analysis. We linked to the Discharge Abstract Database to capture hospital admissions within 14 days of the first positive test. Among those admitted, we also estimated the proportion requiring critical care. For mortality within 90 days of the first SARS-CoV-2 positive test, the date of death was obtained from the RPDB.

### Statistical analysis

Descriptive statistics included N (%) and mean (SD) where appropriate. Logistic regression was used to identify characteristics associated with SARS-CoV-2 infection among physicians, reported by using odds ratios (OR) with 95% confidence intervals (CI). For comparison with community-matched controls, conditional logistic regression was used, adjusted for age as a continuous variable for possible residual confounding. For comparison with general controls, logistic regression was used, adjusted for age, sex, comorbidity score (0, 1, 2+), rurality, and dissemination-area-level quintiles of material deprivation quintile and ethnic diversity (17). The relative risk of death was estimated by using a modified Poisson regression, adjusted for age, sex, comorbidity score, rurality, material deprivation quintile, and ethnic diversity quintile, reported as a risk ratio (RR) with 95% CI (18). In sensitivity analysis, we also adjusted for PCR testing rate by using the number of tests from the previous wave of infection as a surrogate for the testing rate in the index wave for as-yet uninfected individuals. This removed any possibility of reverse causality (eg, higher testing after infection). Among physicians, this sensitivity analysis was performed for the Omicron wave (wave 5 PCR testing rate as a predictor of wave 6 infection), as there were more events for modeling the effect by specialty. For physicians vs controls, this sensitivity analysis was not repeated since access to PCR was markedly different between physicians and controls after wave 5 (19). For comparison between groups for the occurrence of uncommon events (eg, use of critical care, mortality within 14 days of hospitalization), we used Fisher exact test. *P* values <0.05 were considered statistically significant.

### Privacy and software

All analyses were performed at Ontario Health using SAS version 9.4 (SAS Institute Inc., Cary, NC, USA). This study was compliant with section 45 (1) of Personal Health Information Protection Act (PHIPA), Ontario, Canada (Ontario Health is a prescribed entity): ethics review was not required as per the privacy assessment at Ontario Health.

## Results

Of the 37 495 valid physicians identified in the CPDB, 30 617 (80.7%) were linked to a single record in the RPDB and used for analysis (Figure 1). Physicians were mostly male (56%), with a mean age of 47.7 (standard deviation [SD] 13.0) years as of March 1, 2020. Most physicians had no comorbidity (97% with a Charlson score of 0), lived predominantly in an urban area (96%), resided in the least materially-deprived neighborhoods (53% in the least deprived quintile), and resided in neighborhoods with mid-to-moderately high ethnic diversity (54%). The most common physician specialties were family practice/general practice (46%), followed by psychiatry (6.7%), internal medicine (6.1%), and pediatrics (5.1%). A total 1585 (5.2%) of physicians billed LTC codes, the majority of whom were family/general practice (n = 1470), followed by internal medicine (n = 40), psychiatry (n = 23), physical medicine (n = 13), and geriatrics (n = 11).

### SARS-CoV-2 infection among physicians

**Figure 2:**
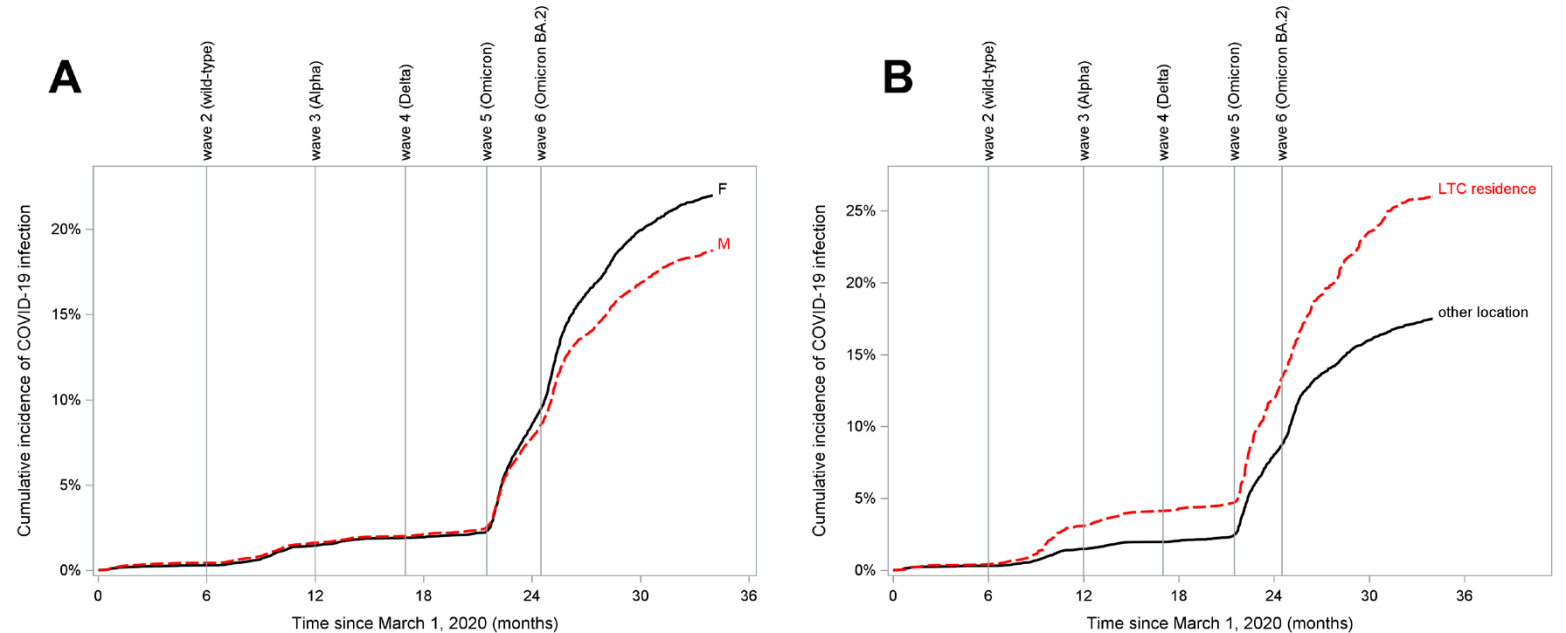
Time until SARS-CoV-2 infection among physicians by sex and location. Cumulative incidence plot showing the time until the first SARS-CoV-2 infection since the start of the COVID-19 pandemic in Ontario stratified by physician sex (**A**) and employment at a long-term care (LTC) facility or equivalent (**B**).

Between March 1, 2020 and December 31, 2022, a total 6172 of 30 617 (20%) physicians tested positive on PCR for SARS-CoV-2. SARS-CoV-2 infection rates were similar between male and female physicians until mid-way through the Omicron wave (Figure 2A), at which point the rates of infection for female physicians began to exceed those for male physicians; this difference continued to grow through to the end of the study period. Physicians working in LTC settings began to test positive for SARS-CoV-2 at significantly higher rates than physicians in other settings starting in the middle of wave 2; this difference continued to grow through to the end of the study period (Figure 2B).

After adjustment for age, sex, comorbidity, rurality, material deprivation, ethnic diversity, LTC status, and specialty, SARS-CoV-2 infection was more likely for female physicians (OR 1.08 [1.02-1.15]) and less likely for older physicians (OR 0.75 [0.74-0.77] per 10 years), rural residents (OR 0.80 [0.68-0.94]), and physicians residing in neighborhoods with the most material deprivation (OR 0.77 [0.66-0.91]) and greatest ethnic diversity (OR 0.79 [0.70-0.89]) (Table 1).

**Table 1:** Physician characteristics

	SARS-CoV-2 negative; n (%) N = 24 445	SARS-CoV-2 positive; n (%) N = 6172	Crude odds ratio	*P* value	Model 1 Adjusted odds ratio*	*P* value	Model 2 Adjusted odds ratio^†^	*P* value
Age, per 10 years	48.5 (13.2)	44.2 (11.7)	0.76 (0.75-0.78)	<0.0001	0.75 (0.74-0.77)	<0.0001	0.78 (0.76-0.81)	<0.0001
Sex								
male	13 996 (57)	3231 (52)	1.0 (ref)	<0.0001	1.0 (ref)	0.01	1.0 (ref)	0.001
female	10 449 (43)	2941 (48)	1.22 (1.15-1.29)		1.08 (1.02-1.15)		1.14 (1.07-1.22)	
Comorbidity								
0	23 725 (97)	6019 (98)	1.0 (ref)	0.03	1.0 (ref)	0.27	1.0 (ref)	0.57
1	629 (2.6)	142 (2.3)	0.89 (0.74-1.07)		1.14 (0.94-1.38)		1.12 (0.90-1.39)	
2+	91 (0.4)	11 (0.2)	0.48 (0.26-0.89)		0.74 (0.39-1.40)		0.85 (0.39-1.86)	
Rurality								
urban	23 099 (95)	5903 (96)	1.0 (ref)	0.001	1.0 (ref)	0.006	1.0 (ref)	<0.0001
rural	1114 (5)	223 (4)	0.78 (0.68-0.91)		0.80 (0.68-0.94)		0.70 (0.59-0.83)	
Material deprivation								
1 (least)	12 550 (52)	3336 (55)	1.0 (ref)	0.001	1.0 (ref)	0.002	1.0 (ref)	0.002
2	5631 (23)	1351 (22)	0.90 (0.84-0.97)		0.90 (0.83-0.96)		0.89 (0.82-0.97)	
3	3185 (13)	799 (13)	0.94 (0.87-1.03)		0.94 (0.86-1.03)		0.97 (0.88-1.08)	
4	1720 (7)	423 (7)	0.93 (0.83-1.04)		0.93 (0.83-1.04)		0.97 (0.85-1.10)	
5 (most)	1012 (4)	206 (3)	0.77 (0.66-0.89)		0.77 (0.66-0.91)		0.74 (0.62-0.89)	
Ethnic diversity								
1 (least)	2467 (10)	573 (9)	1.0 (ref)	<0.0001	1.0 (ref)	<0.0001	1.0 (ref)	0.0002
2	3843 (16)	1014 (17)	1.14 (1.01-1.27)		1.03 (0.91-1.16)		1.08 (0.95-1.23)	
3	5993 (25)	1700 (28)	1.22 (1.10-1.36)		1.06 (0.95-1.19)		1.16 (1.02-1.31)	
4	7087 (29)	1828 (30)	1.11 (1.00-1.23)		0.96 (0.85-1.07)		1.09 (0.96-1.23)	
5 (most)	4708 (20)	1000 (16)	0.92 (0.82-1.03)		0.79 (0.70-0.89)		0.92 (0.80-1.05)	
Long-term-care facility^‡^								
no	23 274 (96)	5758 (93)	1.0 (ref)		1.0 (ref)	<0.0001	1.0 (ref)	0.03
yes	1171 (4.8)	414 (6.7)	1.43 (1.27-1.61)		1.80 (1.59-2.04)		1.16 (1.02-1.32)	
Billing volume								
lowest quintile	3717 (22)	740 (14)	1.0 (ref)	<0.0001	-	-	1.0 (ref)	<0.0001
mid-to-low quintile	3481 (20)	976 (19)	1.41 (1.27-1.57)				1.39 (1.25-1.56)	
middle quintile	3295 (19)	1185 (22)	1.81 (1.63-2.00)				1.85 (1.66-2.06)	
mid-to-high quintile	3237 (19)	1220 (23)	1.89 (1.71-2.10)				2.02 (1.81-2.25)	
highest quintile	3312 (19)	1150 (22)	1.74 (1.57-1.93)				2.05 (1.82-2.30)	
Specialty								
anesthesia	1060 (77)	325 (23)	1.36 (1.19-1.55)		1.48 (1.30-1.70)		0.87 (0.76-1.01)	
cardiology	505 (81)	121 (19)	1.06 (0.87-1.30)		1.22 (0.99-1.50)		0.76 (0.61-0.95)	
cardiothoracic surgery	72 (77)	21 (23)	1.29 (0.79-2.11)		1.57 (0.96-2.57)		0.88 (0.54-1.45)	
clinical immunology	51 (81)	12 (19)	1.04 (0.56-1.96)		0.99 (0.53-1.87)		0.81 (0.33-1.99)	
dermatology	208 (88)	28 (12)	0.60 (0.40-0.89)		0.62 (0.41-0.93)		0.76 (0.47-1.25)	
diagnostic radiology	848 (80)	212 (20)	1.11 (0.95-1.30)		1.24 (1.06-1.46)		0.69 (0.58-0.83)	
emergency medicine	356 (72)	136 (28)	1.70 (1.39-2.08)		1.55 (1.26-1.90)		1.00 (0.81-1.24)	
endocrinology	208 (84)	40 (16)	0.85 (0.61-1.20)		0.86 (0.61-1.22)		0.63 (0.43-0.93)	
family/general practice	11 403 (82)	2570 (18)	1.0 (ref)	<0.0001	1.0 (ref)	<0.0001	1.0 (ref)	<0.0001
gastroenterology	208 (78)	60 (22)	1.28 (0.96-1.71)		1.35 (1.01-1.81)		0.77 (0.57-1.05)	
general surgery	581 (75)	191 (25)	1.46 (1.23-1.73)		1.61 (1.36-1.91)		0.96 (0.80-1.16)	
genetics	31 (69)	14 (31)	2.00 (1.06-3.77)		2.03 (1.07-3.85)		1.36 (0.71-2.61)	
geriatrics	118 (77)	36 (23)	1.35 (0.93-1.97)		1.36 (0.93-1.99)		0.76 (0.51-1.12)	
hematology	162 (70)	68 (30)	1.86 (1.40-2.48)		1.89 (1.41-2.53)		1.08 (0.80-1.45)	
infectious disease	123 (71)	51 (29)	1.84 (1.32-2.56)		1.89 (1.35-2.64)		1.12 (0.80-1.58)	
internal medicine	1460 (78)	417 (22)	1.27 (1.13-1.42)		1.26 (1.12-1.42)		0.87 (0.76-0.99)	
medical oncology	183 (73)	69 (27)	1.67 (1.26-2.21)		1.68 (1.26-2.23)		0.93 (0.70-1.24)	
nephrology	160 (75)	53 (25)	1.47 (1.07-2.01)		1.57 (1.14-2.16)		0.85 (0.61-1.17)	
neurology	387 (80)	94 (20)	1.08 (0.86-1.36)		1.12 (0.89-1.42)		0.75 (0.59-0.96)	
neurosurgery	90 (80)	23 (20)	1.13 (0.72-1.80)		1.25 (0.78-1.99)		0.74 (0.46-1.18)	
nurse practitioners	46 (84)	9 (16)	0.87 (0.42-1.78)		0.95 (0.46-1.96)		0.72 (0.08-6.23)	
obstetrics/gynecology	704 (78)	194 (22)	1.22 (1.04-1.44)		1.32 (1.11-1.56)		0.81 (0.68-0.97)	
ophthalmology	375 (87)	54 (13)	0.64 (0.48-0.85)		0.72 (0.54-0.97)		0.51 (0.38-0.70)	
orthopedic surgery	467 (78)	129 (22)	1.23 (1.00-1.50)		1.31 (1.07-1.61)		0.77 (0.62-0.95)	
other^§^	99 (85)	17 (15)	0.76 (0.46-1.28)		0.87 (0.51-1.46)		0.51 (0.30-0.88)	
otolaryngology	215 (80)	53 (20)	1.09 (0.81-1.48)		1.18 (0.87-1.61)		0.79 (0.57-1.08)	
pediatrics	1153 (73)	418 (27)	1.61 (1.43-1.81)		1.66 (1.47-1.88)		1.25 (1.09-1.44)	
pathology, microbiology, clinical biochemistry	288 (78)	81 (22)	1.25 (0.97-1.60)		1.51 (1.17-1.95)		1.30 (0.97-1.75)	
physical medicine	184 (80)	46 (20)	1.11 (0.80-1.54)		1.11 (0.80-1.55)		0.88 (0.62-1.26)	
plastic surgery	200 (84)	38 (16)	0.84 (0.59-1.20)		0.89 (0.63-1.27)		0.63 (0.44-0.91)	
psychiatry	1674 (82)	372 (18)	0.99 (0.87-1.11)		1.12 (0.99-1.27)		0.84 (0.74-0.97)	
radiation oncology	164 (78)	45 (22)	1.22 (0.87-1.70)		1.34 (0.96-1.88)		0.78 (0.56-1.10)	
respiratory disease	205 (79)	53 (21)	1.15 (0.85-1.56)		1.20 (0.88-1.64)		0.74 (0.54-1.02)	
rheumatology	181 (83)	36 (17)	0.88 (0.62-1.27)		0.93 (0.65-1.34)		0.88 (0.59-1.30)	
urology	220 (77)	65 (23)	1.31 (0.99-1.73)		1.36 (1.02-1.81)		0.81 (0.60-1.08)	
vascular surgery	56 (73)	21 (27)	1.66 (1.01-2.75)		1.64 (0.98-2.76)		0.93 (0.55-1.58)	

*adjusted for age, sex, comorbidity score, long-term care facility status, rurality, material deprivation, ethnic diversity, and specialty.

†adjusted for age, sex, comorbidity score, long-term care facility status, rurality, material deprivation, ethnic diversity, specialty, and the rank order number of billings (surrogate for patient volume).

‡physician was classified as working at a long-term care facility if the physician billed at least 20 instances corresponding to services rendered at such a facility.

§includes critical care medicine, community medicine, nuclear medicine, and thoracic surgery (combined due to small counts).

### SARS-CoV-2 infection by specialty

**Figure 3:**
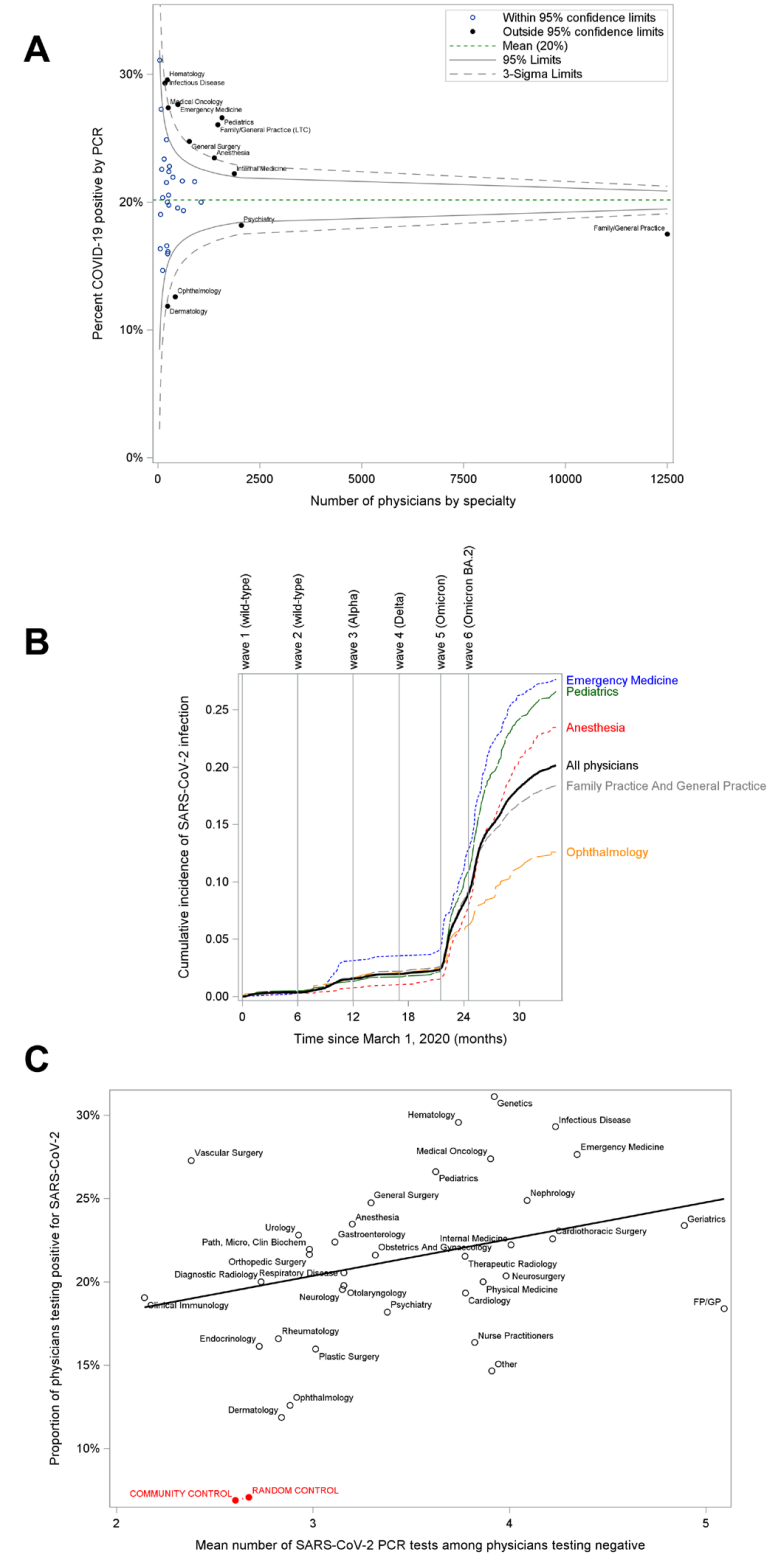
Variation in SARS-CoV-2 infection by specialty. (**A**) Funnel plot for the percent of physicians by specialty testing positive for SARS-CoV-2 on polymerase chain reaction (PCR) as a function of the number of physicians per specialty. (**B**) Cumulative incidence plot showing the time until the first SARS-CoV-2 infection since the start of the COVID-19 pandemic in Ontario stratified by select physician specialties. (**C**) Association between SARS-CoV-2 testing rate and positivity by specialty. Solid line is the regression line for this association (physicians only). FP/GP – family practice/general practice. Testing rate was restricted to physicians who did not test positive to avoid testing bias following a positive test, which would artificially strengthen this observed correlation.

There was significant variation by physician specialty, with lower rates observed among dermatologists (12%), ophthalmologists (13%), and family/general practitioners (18%), and higher rates observed among emergency physicians (28%) and pediatricians (27%) (Table 1; Figure 3). Time until infection followed a similar pattern across specialties, with a large inflection coinciding with the emergence of the Omicron variant (Figure 3B; Supplemental Figure 1(Supplementary figure 1)). For several specialty groups, there was an initial surge of cases mid-way through the second wave (eg, emergency medicine, general surgery, endocrinology).

After adjustment for age, sex, comorbidity, rurality, material deprivation, and ethnic diversity, LTC employment, relative to family/general practitioners, the highest risk of SARS-CoV-2 infection was among hematologists (OR 1.89 [1.41-2.53]), infectious disease specialists (OR 1.89 [1.35-2.64]), medical oncologists (OR 1.68 [1.26-2.23]), pediatricians (OR 1.66 [1.47-1.88]), general surgeons (OR 1.61 [1.36-1.91]), emergency medicine physicians (OR 1.55 [1.26-1.90]), and anesthesiologists (OR 1.48 [1.30-1.70]). After additionally adjusting for patient volume during the pandemic (likely a confounder), the association between specialty and SARS-CoV-2 infection was markedly affected. For most specialties, adjusting for billing volumes during the pandemic abrogated the association relative to family/general practitioners (eg, the CI for the OR for emergency medicine, infectious disease, hematology, medical oncology, etc crossed unity). Pediatricians were the only specialists who remained at a higher risk of infection (OR 1.25 [1.09-1.44] vs family/general practice). For diagnostic radiologists, internists, obstetricians/gynecologists, and orthopedic surgeons, the risk of infection reversed after accounting for billing volumes (becoming less likely relative to family/general practice). Lastly, ophthalmologists were less likely to test positive on PCR for SARS-CoV-2 regardless of adjustment for billing volumes.

Specialties comprised of a higher proportion of physicians testing positive tended to be those with higher PCR testing rates (Figure 3C). To explore whether PCR testing rates accounted for the association between specialty and SARS-CoV-2 infection, we performed a logistic regression for as-yet uninfected persons by the start of wave 6, using their baseline (wave 5) testing rates as a measure of general testing practices. After adjusting for age, sex, comorbidity score, LTC employment, material deprivation, ethnic diversity, billing volume rank (quintiles of billing volume), and SARS-CoV-2 testing rates during wave 5, the relative odds of SARS-CoV-2 infection during wave 6 was highest among pediatricians (OR 1.46 [1.24-1.72]) and pathologists, microbiologists, and clinical biochemists (OR 1.87 [1.34-2.60]) and lowest among ophthalmologists (OR 0.54 [0.36-0.81]) compared with family/general practice (Supplemental Table 3(Supplementary table 3)). SARS-CoV-2 infection was also more likely among physicians with a higher billing volume rank (OR 1.22 [1.18-1.26]) and those with a higher testing rate during wave 5 (OR 1.23 [1.19-1.27]).

### Comparison with non-physicians

**Figure 4:**
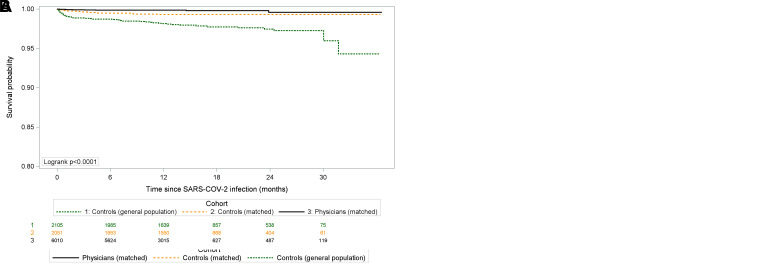
Outcome by cohort. (**A**) Cumulative incidence plot showing the time until the first SARS-CoV-2 infection since the start of the COVID-19 pandemic in Ontario stratified by cohort (n = 29 763 per group); (**B**) Kaplan-Meier plot for all-cause mortality since the first SARS-CoV-2 infection, by cohort.

We compared the likelihood of SARS-CoV-2 infection confirmed by PCR among physicians with community-matched controls and the general population (n = 29 763 physicians were matched to one control) (Figure 4A; Supplemental Table 2 (Supplementary table 2)for cohort characteristics). During wave 1, physicians had a higher risk of infection compared with community-matched controls (OR 2.34 [1.65-3.32]) and general population controls (OR 1.62 [1.15-2.26]) (Table 2). By the end of the third wave and unchanged through the end of the fourth, the risk of infection among physicians was similar to community-matched controls (OR 0.93 [0.83-1.05], *P* = 0.23) but lower than for general population controls (OR 0.74 [0.66-0.83], *P* < 0.0001).

**Table 2:** SARS-CoV-2 infection by population over time

	No. (%) of						
	physicians	community-matched controls	general population controls	Physician vs community-matched controls		Physician vs frequency-matched general population controls	
	N = 29 763	N = 29 763	N = 29 763	odds ratio (95% confidence interval)*	*P* value	odds ratio (95% confidence interval)^†^	*P* value
Wave 1	108 (0.36)	41 (0.16)	67 (0.23)	2.34 (1.65-3.32)	<0.0001	1.62 (1.15-2.26)	0.005
Wave 1-2	457 (1.54)	332 (1.12)	462 (1.55)	1.38 (1.20-1.59)	<0.0001	1.04 (0.90-1.20)	0.57
Wave 1-3	582 (1.96)	623 (2.09)	824 (2.77)	0.93 (0.83-1.05)	0.23	0.74 (0.66-0.83)	<0.0001
Wave 1-4	705 (2.37)	764 (2.57)	944 (3.17)	0.92 (0.83-1.02)	0.12	0.78 (0.70-0.87)	<0.0001

*conditional logistic regression to accommodate matching.

†logistic regression adjusted for age, sex, comorbidity, rurality, and neighborhood material deprivation and ethnic diversity quintiles.

### Outcomes after infection

Within 14 days of the first positive SARS-CoV-2 PCR test, 63/6010 (1.05%) physicians, 65/2051 (3.17%) matched controls, and 113/2105 (5.37%) general population controls were hospitalized (*P* < 0.0001; Fischer’s exact test). Compared with physicians and after adjustment for sociodemographic characteristics and comorbidity score, the relative risk (RR) of admission was 2.78 (1.97-3.93) for the matched control group and 3.56 (2.55-4.98) for the general population control group. Among those admitted, 11/63 (17%) physicians, 15/65 (23%) community controls, and 29/113 (26%) general population controls required ICU admission during their hospitalization (*P* = 0.48; Fisher exact test).

The survival rate after SARS-CoV-2 infection was high in all three cohorts but higher among physicians (log-rank *P* < 0.0001), followed by the community-matched control group and the general population control group (Figure 4B). Within 90 days of SARS-CoV-2 infection, 7/6010 (0.12%) physicians, 8/2051 (0.39%) matched controls, and 24/2105 (1.14%) general population controls died due to any cause (*P* < 0.0001; Fischer’s exact test for all three groups; *P* = 0.03 for only physicians vs community-matched controls). Restricted to patients hospitalized within 14 days of the SARS-CoV-2 positive test, there was no difference in mortality between the groups (*P* = 0.15; Fisher exact test; output suppressed due to small counts).

## Discussion

In this retrospective cohort study, pediatricians and physicians practicing in LTC facilities were more likely to test positive for SARS-CoV-2, independent of patient volume.

Previous studies examining SARS-CoV-2 infection among health care providers were limited to the early phases of the pandemic. One study in Ontario demonstrated higher infection rates, but lower risk of death, among health care workers compared with non-health care workers during the first wave of the pandemic (9). One study in Northern Italy demonstrated increased mortality among physicians and dentists in 2020, but did not consider population controls (20). In one study in Mexico City, health care workers comprised a significant proportion of persons testing positive for SARS-CoV-2 between February and August 2020, but had lower mortality (10). In a large academic health care system in the United States, patient-facing health care workers were more likely to test positive for SARS-CoV-2, but did not have higher rates of admission (11,12). Our findings support these early international observations, but we extended the study scope beyond the wild-type variant (first two waves). During the early pandemic, higher rates of infection among physicians may have been driven by differential access to PCR testing compared with community-matched controls and the general population (19). The lower risk of hospitalization implicates the possible effect of non-discriminant testing among physicians. We report a lower rate of infection among physicians compared with the general population since wave 3 (Alpha variant) and a similar risk compared with community-matched controls. Early efforts to prioritize vaccination and access to high-quality personal protective equipment for physicians may have driven this effect. Despite being in a high-risk setting (higher presumed risk of exposure) and having immediate access to PCR testing (higher presumed risk of testing bias), physicians did not have a higher risk of infection compared with community-matched controls since wave 3.

SARS-CoV-2 infection varied by specialty, which is in part likely due to in-person patient volumes and different testing rates. In 2020 (the first nine months of the pandemic), emergency physicians, infectious disease physicians, and family physicians had the highest rates of SARS-CoV-2 testing (>47%), with the highest rates of positivity among internists (1.3%), postgraduate medical trainees (1.1%), and family physicians (0.9%) (the denominator includes those not tested) (21). Proclivity toward testing could be due to presence of symptoms, hospital-specific protocols, specialty-specific protocols, testing eligibility, or physician preference. Specialists can be considered working in high-risk settings due to high patient volume, which is distinct from working in settings where transmission is more likely (eg, masking children and infants effectively is challenging). To account for this, we presented two models: one adjusted for sociodemographic characteristics alone and one additionally adjusted for patient volume. Our findings suggest that, for example, the higher risk of exposure among emergency physicians and infectious disease specialists may be due to predominantly higher patient volumes, but this was not the case for pediatricians.

Lower hospitalization among physicians compared with community and population controls may suggest that more mild cases were being captured among physicians rather than a true difference in outcomes. This may be due to earlier detection or early vaccination efforts prioritizing physicians (particularly those in higher-risk setting or working with more vulnerable patient populations). Although data from Ontario are lacking, the uptake of the SARS-CoV-2 vaccination among health care workers in North America was higher (85.6%) than in Asia (79.5%), Europe (72.8%), and Africa (65.6%) (22). One study from the United States suggested that personal protective equipment use, vaccine requirements, infection prevention protocols, adequate staffing, and other workplace-based protective measures were effective in preventing excess mortality among physicians (23). However, in our study, after restricting to the most severe infections (eg, those resulting in hospitalization), there was no difference in mortality between physicians and controls. The difference in mortality due to longer follow-up (eg, 90-day all-cause mortality) is likely due to confounding unaccounted for due to small event size.

Before the Omicron wave (wave 2 through the Delta wave), access to PCR testing was not restricted to physicians, but physicians may have had easier access compared with the general population, particularly as vaccine rollout began in early 2021 (24). Despite this, physicians did not have a higher rate of positivity compared with community-matched controls. During the Omicron wave, however, access to PCR was more restrictive, and as the general population may have relied more on rapid antigen testing (or not tested at all), testing bias prevents any meaningful comparison between physicians and controls since wave 5 (19). Thus, while conclusions about physicians relative to non-physician populations are subject to strong ascertainment bias since the Omicron era (and were therefore not reported), descriptive statistics between physician specialties remain valuable. However, throughout the pandemic, different institutions and specialties may have had different protocols for SARS-CoV-2 asymptomatic screening and return-to-work policy.

The effect of SARS-CoV-2 positivity among physicians has had significant human resource implications. Such direct effects have strained the health care system and have been compounded by infection-related absenteeism among nurses and other allied health professionals in high-risk settings, who are critical to ongoing health care in the province. One study demonstrated that the rate of infection was significantly higher among nurses (1129 per 100 000) than physicians (475.3 per 100 000) (9). Without data on other staffing groups, the number of whom greatly surpasses that of physicians, the overall direct impact of the pandemic on human resources is underestimated.

The increased likelihood of contracting SARS-CoV-2 infection among female physicians persisted after adjusting for specialty and other sociodemographic factors. This could be due to both professional and personal differences. In the professional sphere, some studies have reported that female physicians spend more time with patients than their male counterparts (25). This may increase the risk of infection. More generally, however, women may be more likely than men to contract SARS-CoV-2 in the community owing to higher involvement in care work at home (eg, childcare and tending to sick family members) (26).

### Limitations

The greatest limitation of this study is testing bias. Although we attempted to account for this by adjusting for baseline testing rates (using the previous wave testing rate as a surrogate), this is unlikely to fully account for the differential access to testing among physicians. Another limitation is the dependence on OHIP billing to identify and link physicians. There are therefore some physicians who were missed from the study. We were unable to identify postgraduate medical trainees, a group with a high positivity rate in the early pandemic (21). Another limitation is the change in behaviors and practices throughout the pandemic. Physicians may have been redeployed to offload the burden of practicing physicians during times of staff shortages, resulting in some misclassification of specialty (eg, a medical oncologist recruited to help in a different service area). Moreover, some physicians may have adopted virtual care options for some or all of their patients. Physicians in certain specialties may have had greater interactions with patients with SARS-CoV-2 infection (known or unknown) than others, and adjusting for patient volume may not account for this. Moreover, compliance with standard precaution measures remains uncontrolled.

As another limitation, physicians may have started work mid-way through the pandemic, but we lacked granularity on the physicians’ start date. For new physicians, we assumed the physician was practicing as a resident beforehand, and exposure risk did not change drastically. For retired physicians rejoining the work-force, we expect this to partially contaminate the physician group and reduce the measures of effect toward unity (eg, to have a conservative effect). We also lacked granularity on physician end-dates. Some physicians may have retired or stopped working during the pandemic, but this is also expected to have a conservative effect on the measures of association in this study.

One limitation is the assumption of whether physicians contracted SARS-CoV-2 in the workplace vs the community. In the first three waves of the pandemic, closures and public fear limited person-to-person contact in the community. Thus, in these waves we assume that the higher rate of infection among physicians was related to employment rather than community-derived infection. This assumption may not hold for later waves as public health restrictions in Ontario began to ease following wave 3. However, a higher billing volume was significantly associated with a higher risk of infection and adjusting for billing volume reduced the variability in the risk of infection across specialties. This supports the notion of workplace-derived infection rather than community-derived infection.

Another limitation is the lack of negative PCR tests following a positive PCR test (21). Anecdotally, facilities required differing degrees of evidence of SARS-CoV-2 negativity before resuming work, ranging from a negative PCR test to relief of symptoms after 10 days. We therefore excluded recurrent infections.

### Implications and conclusions

For preparedness planning for future outbreaks of SARS-CoV-2 or a new pandemic, our results suggest that there are several specialty physician groups that may be at higher risk of workplace infection that may be candidates for additional safety measures. These include high-volume physicians who work in settings where patient contact is unavoidable (eg, emergency physicians). Examples include efforts to encourage patients to seek virtual care for non-urgent care. Physicians working in settings where patients may be unable to wear personal protective equipment effectively (eg, infants) may be prioritized for vaccination whenever possible. For lower-risk settings (eg, dermatology and ophthalmology), it may be possible to preserve some in-person care without jeopardizing the safety of the physician.

Our study found that physicians had higher rates of infection than community-matched controls during the first two waves of the pandemic in Ontario, but this difference seemed to disappear from wave 3 onward. Physicians practicing in LTC facilities and pediatricians were more likely to test positive on PCR for SARS-CoV-2 than other physicians.
