# Supplementary figures and images for: SARS-CoV-2 infection among physicians over time in Ontario, Canada: a population-based retrospective cohort study
Source: Croat Med J. 2024 Feb;65(1):30–42. doi: 10.3325/cmj.2024.65.30 (PMC10915769; doi:10.3325/cmj.2024.65.30)

Supplemental Figure 1: Cumulative incidence of SARS-CoV-2 infection by specialty

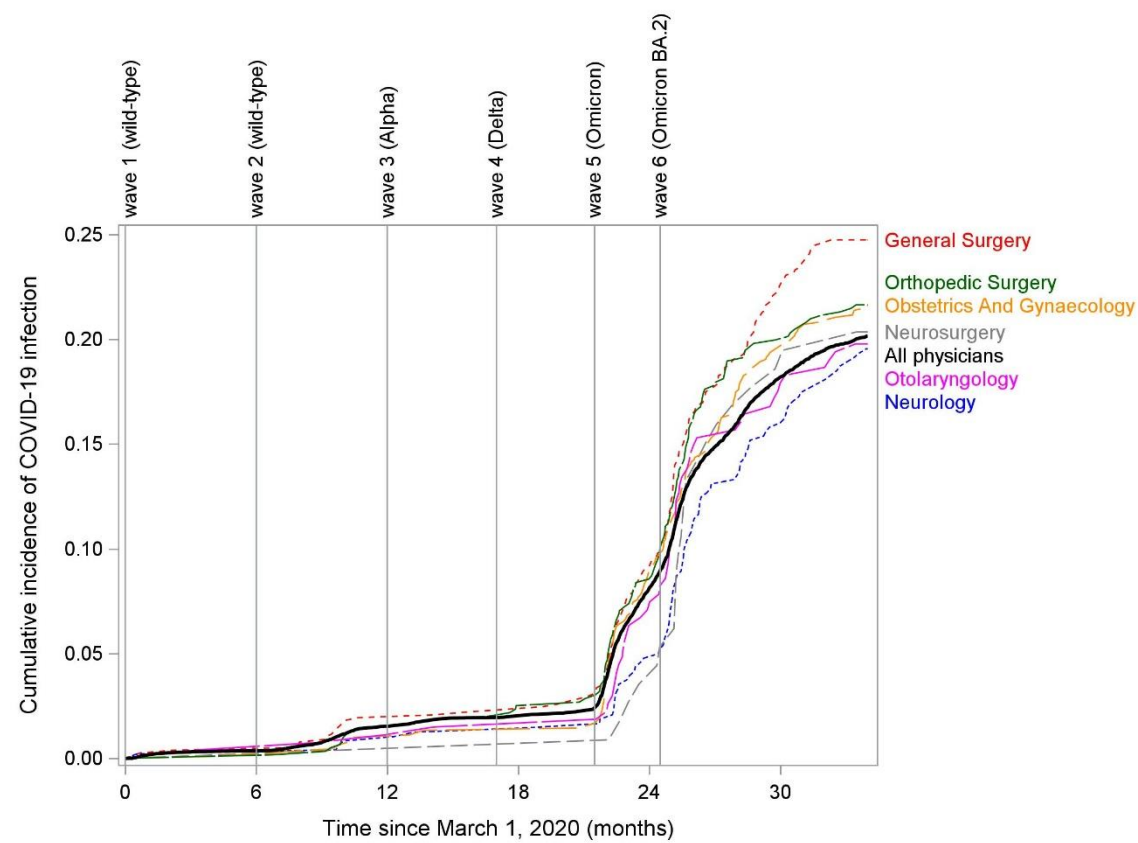

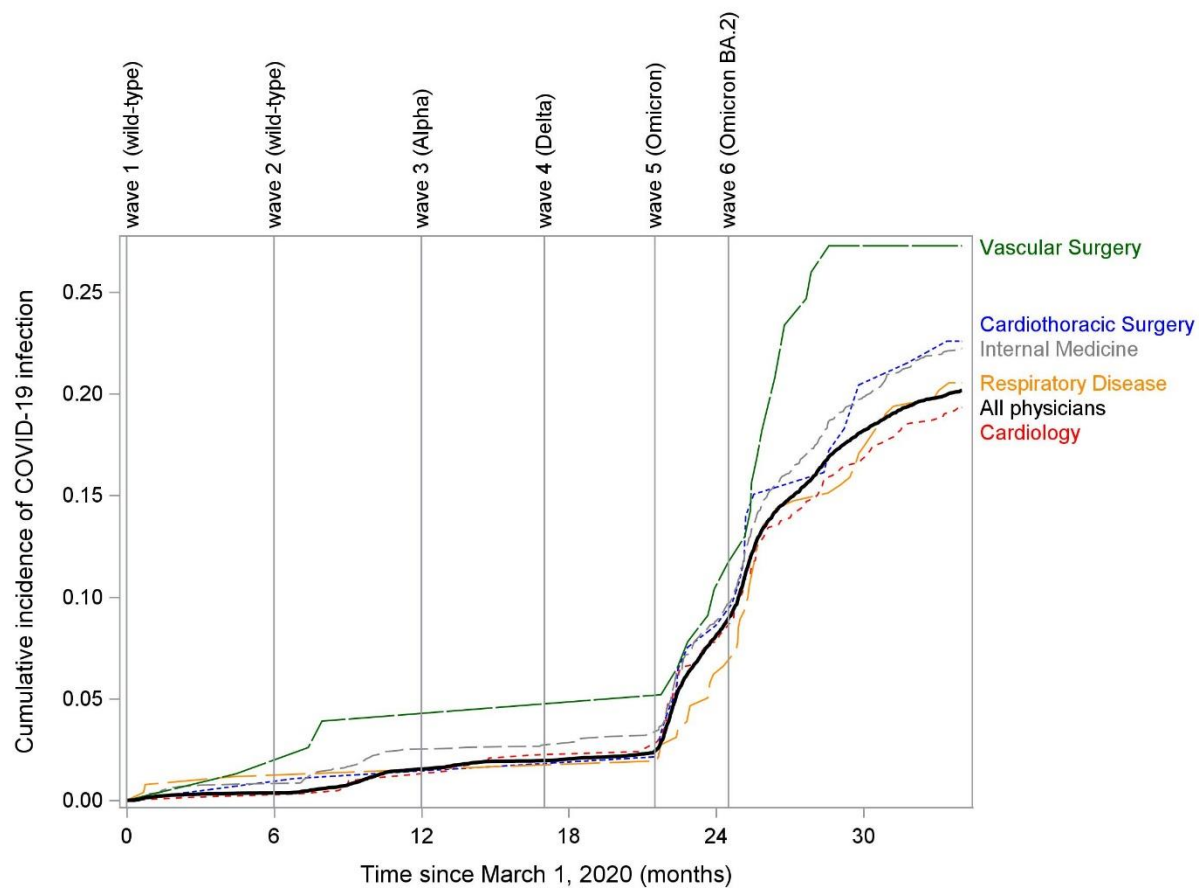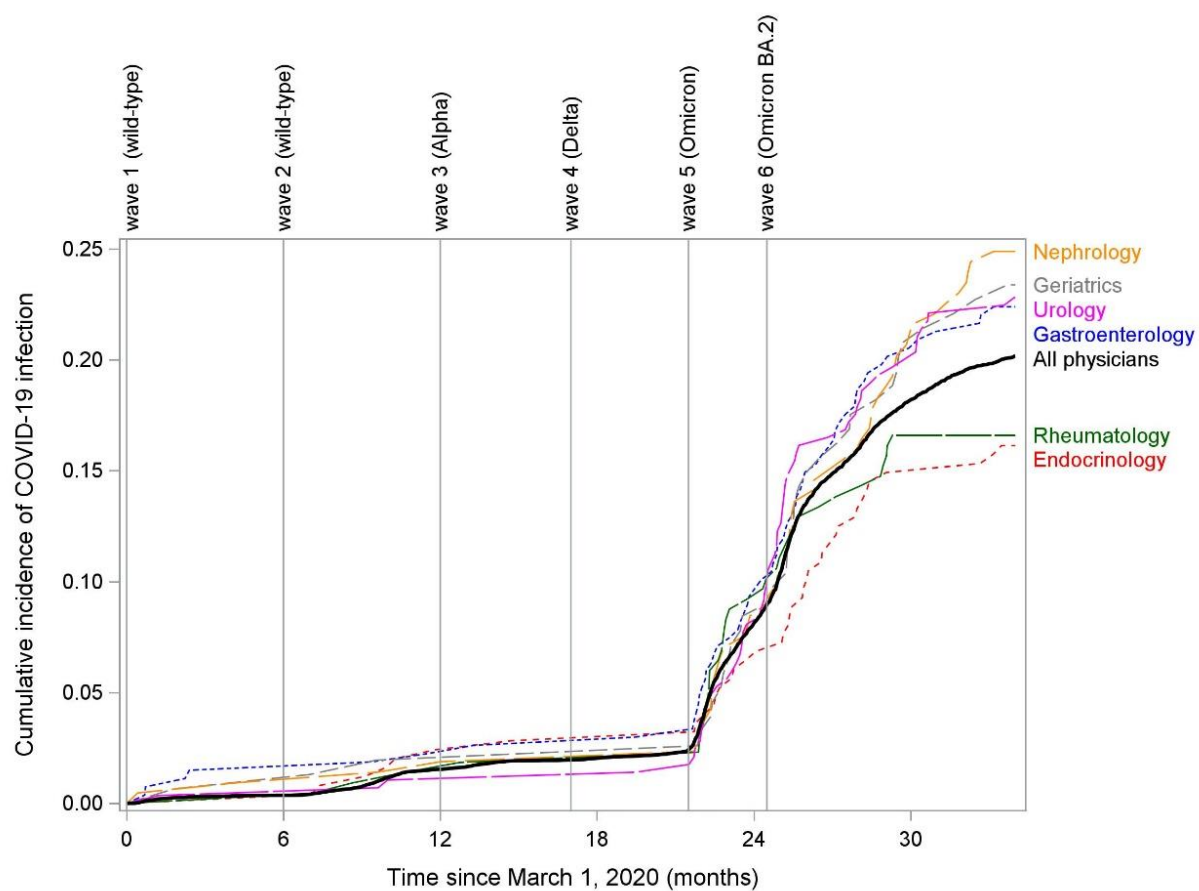

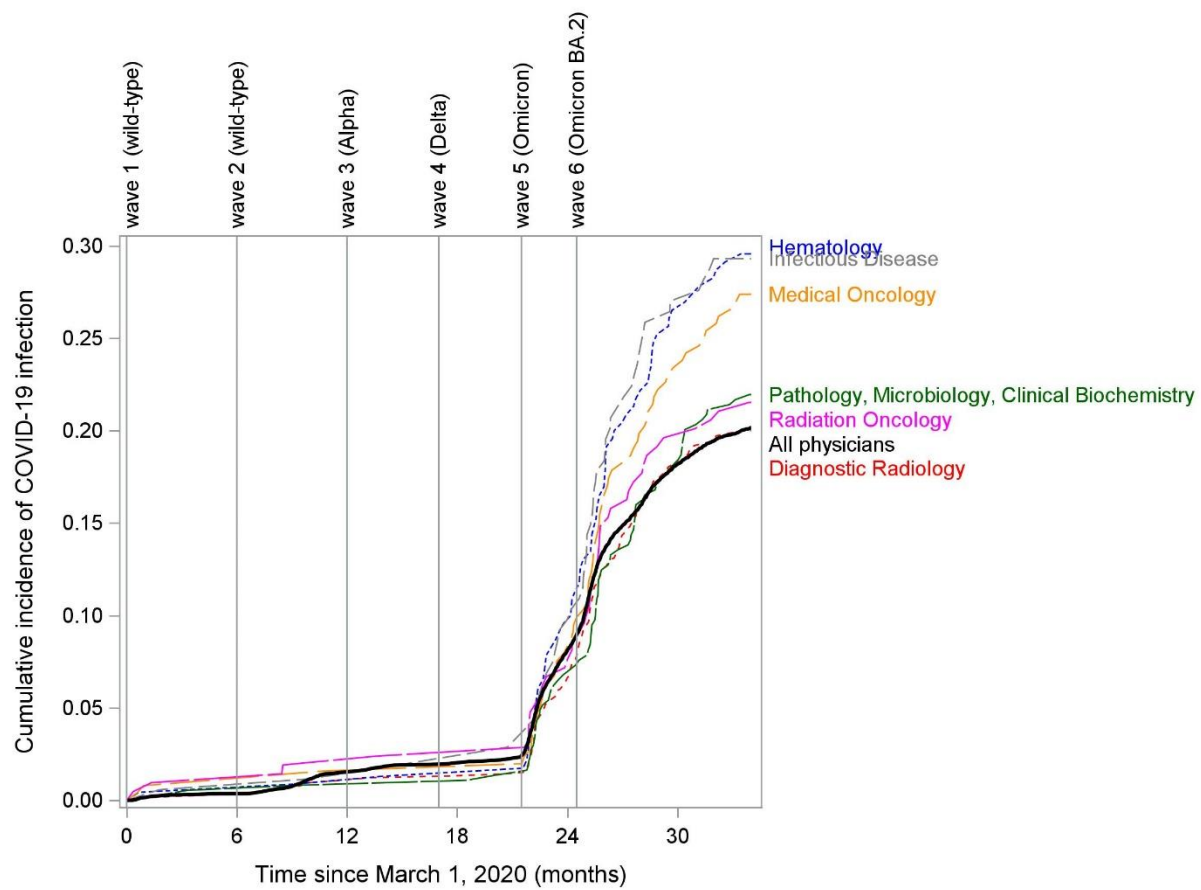

Supplement: Supplementary Table 1 [file CroatMedJ_65_s001.pdf]
