# Supplementary material for: SARS-CoV-2 infection among physicians over time in Ontario, Canada: a population-based retrospective cohort study
Source: Croat Med J. 2024 Feb;65(1):30–42. doi: 10.3325/cmj.2024.65.30 (PMC10915769; doi:10.3325/cmj.2024.65.30)
Supplement: Supplementary Table 1 [file CroatMedJ_65_s002.pdf]

Supplemental Table 1: Algorithm to identify physician health card number

**N physicians yet to be matched**	**Matching Step and Description**	**Number 1:1 matches**	**Duplicates**<br>• **The same physician matches to >1 HIN; or**<br>• **The same HIN matches to >1 physician (duplicates may occur); or**<br>• **Both**
		**N unique physicians**	**N unique physicians**
37,945 (100%)	1. Exact first name, middle name, last name, sex, city	18,432	432
19,081 (50.3%)	2. Exact first name, last name, sex, and city, but middle name is missing from 1 source	2,011	250
16,820 (44.3%)	3. Exact first name, last name, sex, city, but first and last name are reversed and middle name is either the same or missing from 1 source	23	7
16,790 (44.2%)	4. Exact first name, middle name, last name, sex, but ignoring city	7,388	3081
6,321 (16.7%)	5. Exact first name, last name, sex, city, and first 3 digits of middle name or first 1 digit of middle name if only the middle initial is provided from either source	946	7
5,368 (14.1%)	6. First 3 digits of first name, first 3 digits of last name, sex, city, and first 3 digits of middle name or first 1 digit of middle name if only the middle initial is provided from either source	540	218
4,610 (12.1%)	7. Exact first name and last name, sex, and first 3 digits of middle name or first 1 digit of middle name if only the middle initial is provided from either source, and ignoring city	467	168
3,975 (10.5%)	8. Exact first name and last name (REVERSED), sex, and first 3 digits of middle name or first 1 digit of middle name if only the middle initial is provided from either source, and ignoring city	3	2
3,970 (10.5%)	9. Exact first and middle name (REVERSED), exact last name, sex, city	58	0
3,912 (10.3%)	10. Exact first and middle name (REVERSED), exact last name, and sex (ignore city)	37	12
3,863 (10.2%) unmatched	- No more attempted algorithms yielded accurate matches	29,905 matched 1:1 (78.8%)	4,177 (11.0%) matched 1:n or n:n
Clean-up	- De-duplicated records where the same physician was matched to the same HIN multiple times (e.g. since the physician had multiple addresses in Corporate Provider Database)<br>- Remove those whose travel distance is >50km<br>- Only keep remaining 1:1 matches (1 physician matched to only 1 HIN and that HIN is not matched to any other physician)		
	N=30,617 physicians linked and available for analysis		
