# Supplementary material for: SARS-CoV-2 infection among physicians over time in Ontario, Canada: a population-based retrospective cohort study
Source: Croat Med J. 2024 Feb;65(1):30–42. doi: 10.3325/cmj.2024.65.30 (PMC10915769; doi:10.3325/cmj.2024.65.30)
Supplement: Supplementary Table 2 [file CroatMedJ_65_s003.pdf]

Supplemental Table 2: Cohort characteristics by SARS-CoV-2 status

	Physicians (matched) N=29,763			Controls (matched) N=29,763			General population N=29,763		
**SARS-CoV-2 status**	**Negative (n=23,753)**	**Positive (n=6,010)**	**OR (95% CI)[a]**	**Negative (n=27,712)**	**Positive (n=2,051)**	**OR (95% CI)[a]**	**Negative (n=27,658)**	**Positive (n=2,105)**	**OR (95% CI)[a]**
Age	48.4 (13.1)	44.2 (11.6)	0.75 (0.75-0.77)	47.8 (13.0)	44.9 (12.3)	0.84 (0.81-0.87)	47.7 (13.6)	45.0 (12.9)	0.85 (0.82-0.89)
Sex									
Male	13,566 (57%)	3,148 (52%)	1.0 (ref)	15,703 (57%)	1,011 (49%)	1.0 (ref)	15,711 (57%)	1,003 (48%)	1.0 (ref)
Female	10,187 (43%)	2,862 (48%)	1.03 (0.97-1.09)	12,009 (43%)	1,040 (51%)	1.18 (1.08-1.30)	11,947 (43%)	1,102 (52%)	1.26 (1.15-1.38)
Charlson comorbidity									
0 (no hospital record)	13,187 (56%)	2,963 (49%)	1.0 (ref)	15,298 (55%)	852 (42%)	1.0 (ref)	16,610 (60%)	901 (43%)	1.0
0 (hospital record)	9,785 (41%)	2,901 (48%)	1.35 (1.27-1.43)	11,564 (42%)	1,122 (55%)	1.79 (1.63-1.96)	9,196 (33%)	977 (46%)	1.92 (1.74-2.11)
1	653 (3%)	130 (2%)	1.29 (1.06-1.57)	721 (3%)	62 (3%)	2.00 (1.52-2.63)	1,257 (5%)	158 (8%)	2.68 (2.23-3.23)
2+	128 (<1%)	16 (<1%)	1.01 (0.60-1.71)	129 (<1%)	15 (<1%)	3.13 (1.81-5.41)	595 (2%)	69 (3%)	2.88 (2.20-3.77)
Rurality									
Urban	22,575 (95%)	5,775 (96%)	1.0 (ref)	26,381 (95%)	1,969 (67%)	1.0 (ref)	24,737 (90%)	1,942 (93%)	1.0 (ref)
Rural	1103 (5%)	218 (4%)	0.77 (0.66-0.91)	1246 (5%)	75 (4%)	0.88 (0.68-1.14)	2,871 (10%)	153 (7%)	0.74 (0.61-0.90)
Material deprivation									
1 (least)	12,292 (52%)	3,271 (55%)	1.0 (ref)	14,535 (53%)	1,028 (50%)	1.0 (ref)	6,260 (24%)	461 (22%)	1.0 (ref)
2	5,498 (23%)	1,314 (22%)	0.89 (0.83-0.96)	6,345 (23%)	467 (23%)	1.03 (0.92-1.15)	5,336 (20%)	397 (19%)	1.04 (0.91-1.20)
3	3,118 (13%)	782 (13%)	0.93 (0.85-1.02)	3,602 (13%)	298 (15%)	1.15 (1.00-1.32)	4,838 (19%)	396 (19%)	1.16 (1.01-1.34)
4	1,680 (7%)	412 (7%)	0.93 (0.82-1.04)	1925 (7%)	167 (8%)	1.20 (1.01-1.43)	4,766 (18%)	378 (18%)	1.10 (0.96-1.28)
5 (most)	989 (4%)	204 (3%)	0.78 (0.66-0.91)	1113 (4%)	80 (4%)	0.98 (0.77-1.24)	4,856 (19%)	443 (21%)	1.19 (1.04-1.37)
Ethnic diversity									
1 (least)	2,423 (10%)	563 (9%)	1.0 (ref)	2,813 (10%)	173 (8%)	1.0 (ref)	3,974 (15%)	238 (11%)	1.0 (ref)
2	3,758 (16%)	991 (17%)	1.05 (0.66-0.91)	4,466 (16%)	283 (14%)	1.04 (0.85-1.27)	4,193 (16%)	287 (14%)	1.10 (0.92-1.32)
3	5,871 (25%)	1,660 (28%)	1.08 (0.96-1.21)	7,004 (25%)	527 (26%)	1.22 (1.01-1.48)	4,479 (17%)	384 (19%)	1.39 (1.16-1.66)
4	6,291 (29%)	1,789 (30%)	0.98 (0.87-1.10)	8,094 (29%)	616 (30%)	1.25 (1.03-1.50)	5,606 (22%)	465 (22%)	1.36 (1.14-1.62)
5 (most)	4,604 (19%)	980 (16%)	0.80 (0.71-0.90)	5,143 (19%)	441 (22%)	1.39 (1.14-1.69)	7,804 (30%)	701 (34%)	1.46 (1.23-1.73)

[a] adjusted for all variables shown
OR – odds ratio; CI – confidence interval
