# Supplementary material for: SARS-CoV-2 infection among physicians over time in Ontario, Canada: a population-based retrospective cohort study
Source: Croat Med J. 2024 Feb;65(1):30–42. doi: 10.3325/cmj.2024.65.30 (PMC10915769; doi:10.3325/cmj.2024.65.30)
Supplement: Supplementary Table 3 [file CroatMedJ_65_s004.pdf]

Supplemental Table 3: Factors associated with SARS-CoV-2 infection in wave 6

	Adjusted OR (95% CI)[a]	p-value
Age, per 10 years	0.81 (0.78-0.83)	<.0001
Sex		
Male	1.0 (ref)	<.0001
Female	1.21 (1.11-1.31)	
Comorbidity score		
0	1.0 (ref)	0.10
1	1.26 (1.02-1.57)	
2+	0.99 (0.60-1.64)	
Rurality		
Urban	1.0 (ref)	<.0001
Rural	0.60 (0.48-0.75)	
Material deprivation		
1 (least)	1.0 (ref)	0.15
2	0.92 (0.84-1.02)	
3	1.00 (0.89-1.13)	
4	0.95 (0.81-1.11)	
5 (most)	0.78 (0.63-0.98)	
Ethnic diversity		
1 (least)	1.0 (ref)	0.0004
2	1.05 (0.90-1.23)	
3	1.11 (0.95-1.29)	
4	1.03 (0.88-1.20)	
5 (most)	0.83 (0.70-0.98)	
Long-term care facility		
No	1.0 (ref)	0.91
Yes	0.99 (0.84-1.17)	
Billing volume rank, continuous	1.22 (1.18-1.26)	<.0001
Testing rate during wave 5	1.23 (1.19-1.27)	<.0001
Specialty		
Anesthesia	1.17 (0.99-1.39)	
Cardiology	0.74 (0.56-0.98)	↓
Cardiothoracic surgery	0.93 (0.51-1.71)	
Clinical immunology	0.81 (0.24-2.73)	
Dermatology	0.98 (0.54-1.76)	
Diagnostic radiology	0.77 (0.63-0.96)	↓
Emergency medicine	1.06 (0.81-1.39)	
Endocrinology	0.77 (0.48-1.23)	
Family/general practice	1.0 (ref)	<.0001
Gastroenterology	0.79 (0.53-1.16)	

General surgery	1.09 (0.88-1.36)	
Genetics	1.86 (0.89-3.89)	
Geriatrics	0.82 (0.51-1.33)	
Hematology	1.22 (0.86-1.74)	
Infectious disease	1.34 (0.90-2.00)	
Internal medicine	0.88 (0.75-1.04)	
Medical oncology	1.09 (0.78-1.53)	
Nephrology	1.06 (0.73-1.55)	
Neurology	1.08 (0.82-1.43)	
Neurosurgery	1.01 (0.60-1.71)	
Nurse practitioners	1.14 (0.13-9.88)	
Obstetrics/gynecology	0.84 (0.67-1.05)	
Ophthalmology	0.54 (0.36-0.81)	↓
Orthopedic surgery	0.78 (0.59-1.02)	
Other[b]	0.64 (0.35-1.20)	
Otolaryngology	0.92 (0.63-1.35)	
Pediatrics	1.46 (1.24-1.72)	↑
Pathology, microbiology, clinical biochemistry	1.87 (1.34-2.60)	↑
Physical medicine	0.90 (0.58-1.40)	
Plastic Surgery	0.74 (0.47-1.14)	
Psychiatry	1.01 (0.85-1.19)	
Respiratory disease	0.96 (0.67-1.39)	
Rheumatology	0.75 (0.43-1.29)	
Therapeutic radiology	0.84 (0.55-1.28)	
Urology	0.91 (0.64-1.30)	
Vascular surgery	1.05 (0.56-1.99)	

[a] adjusted for all variables in the table

[b] includes critical care medicine, community medicine, nuclear medicine, and thoracic surgery (combined due to small counts)

OR – odds ratio; CI – confidence interval; ↑/↓ indicate higher/lower than the reference category (for ease of visual inspection of the table)
